# Efficacy and safety of acupuncture for cognitive impairment in Alzheimer's disease: a systematic review and meta-analysis

**DOI:** 10.3389/frdem.2024.1380221

**Published:** 2024-07-03

**Authors:** Ruyue Guo, Xiaoming Shen, John Ealing, Jiao Zhou, Jin Lu, Yunfan Ning

**Affiliations:** ^1^Department of Encephalopathy, The First Affiliated Hospital of Henan University of Traditional Chinese Medicine, Zhengzhou, China; ^2^The First Clinical Medical College of Henan University of Chinese Medicine, Zhengzhou, China; ^3^Salford Royal Hospital, Salford, United Kingdom

**Keywords:** Alzheimer's disease, cognitive dysfunction, acupuncture, curative effect, meta-analysis

## Abstract

**Objective:**

To systematically evaluate the efficacy of acupuncture in the treatment of cognitive impairment in Alzheimer's disease (AD) by meta-analysis, in order to provide evidence-based evidence for the application of acupuncture therapy in the clinical process of AD.

**Methods:**

From the establishment of the database to December 31, 2022, China Biomedical Literature Database (CBM), China National Knowledge Network (CNKI), VIP database, WanFang Database, Pubmed, Embase and Cochrane Library Database were systematically searched. To collect published randomized controlled clinical trials (RCTS) of acupuncture in the treatment of cognitive impairment in AD. The subjects in the intervention group were given acupuncture alone or combined with other treatments the same as the control group; the control group received conventional Western medicine treatment. The main outcome indicators of the study were cognitive function assessment of subjects, including: Simple Mental State Examination Scale (MMSE), Assessment of daily Living Ability Scale (ADL), Alzheimer's Disease Cognitive Function Assessment Scale (ADAS-Cog), TCM syndrome score (SDSD), Montreal Cognitive Test (MoCA), Secondary outcome indicators were the occurrence of adverse reactions. Literature screening, data extraction, and quality evaluation of the included literature were performed independently by two researchers, according to bias risk assessment tools recommended in the Cochrane manual. Data were analyzed by RevMan5.3 software. Dichotomous variables were represented by risk ratio (OR) and 95% CI, and continuity variables were represented by mean difference (MD) and 95% CI. For heterogeneity analysis, when *P* > 0.1 and *I*^2^ ≤ 50%, fixed effect model was applied. When *P* ≤ 0.1 and *I*^2^ > 50%, the random effects model is applied.

**Results:**

A total of 1,172 eligible subjects were included in 18 RCTS, including 595 in the intervention group and 577 in the control group. The results of meta-analysis are as follows: acupuncture intervention group improved MMSE [MD = 1.67, 95% CI (0.94, 2.41), *P* < 0.00001], ADL [MD = −1.18, 95% CI (−3.09, 0.72), *P* = 0.22], ADAS-Cog [MD = 3.31, 95% CI (5.84, 0.78), *P* = 0.01], SDSD [MD = 2.40, 95% CI (3.53, 1.26), *P* < 0.0001], MoCA [MD = 4.80, 95% CI (3.74, 5.86), *P* = 0.04] were better than the control group. No serious adverse reactions related to acupuncture were observed in the intervention group, and the incidence and severity of adverse reactions were lower than those in the control group, with statistical significance [OR = 0.17, 95% CI (0.04, 0.67), *P* = 0.01].

**Conclusion:**

Existing data show that acupuncture therapy has certain advantages in improving cognitive dysfunction and improving self-care ability of patients with Alzheimer's disease. However, due to the small number of RCTS and cases evaluating the efficacy of acupuncture, and the possibility of measurement bias and selectivity bias in included studies, it is still unable to conduct high-intensity demonstration on its effectiveness. Further large-scale, high-quality randomized, double-blind controlled trials are needed to evaluate its efficacy.

**Systematic Review Registration:**

https://inplasy.com/inplasy-2021-12-0125/, identifier: INPLASY2021120125.

## 1 Introduction

Alzheimer's disease (AD) is a common neurological degenerative disease in clinical practice, accounting for 80% of the confirmed cases of dementia (Weller and Budson, 2018) and its characteristic progressive cognitive impairment is an important cause of affecting patients' daily life activities, leading to dependence, disability and death (Soria Lopez et al., 2019). It brings a heavy burden to patients, families, society and medical care. According to the World Alzheimer's Disease Report 2018, there are 6 million AD patients in China, which is the country with the largest number of AD patients, and the number of AD patients in China is expected to exceed 40 million by 2050 (GBD 2016 Dementia Collaborators, 2019). However, there is no strong evidence that systematic and targeted treatment strategies can prevent the occurrence and development of AD cognitive impairment (Alzheimer's Association, 2015). The commonly used acetylcholinesterase inhibitors and aspartic acid receptor inhibitors have only partial symptom improvement effect, and the effect is limited (Chen et al., 2020). However, studies (Langa and Levine, 2014) have shown that early diagnosis and treatment of Alzheimer's disease and mild cognitive impairment play a key role in improving the prognosis of patients. Functional near-infrared spectroscopy has been used by some researchers to prove that acupuncture treatment causes changes in the temporal characteristics of hemodynamic responses in patients with mild cognitive impairment, and the classification by image features also reflects a similar trend, indicating that acupuncture can be used in the treatment of patients with mild cognitive impairment (Khan et al., 2022).

AD belongs to the category of “stupidness” and “depression syndrome” in traditional Chinese medicine. Its etiology and pathogenesis mainly include deficiency of pyeonghai, Qi stagnation and blood stasis, phlegm turbidity and obscuring the body. At present, there are relevant literatures verifying the efficacy and safety of acupuncture in the treatment of cognitive impairment in AD. All of these findings suggest that acupuncture therapy has a positive effect on improving cognitive function in patients with mild cognitive impairment. Therefore, acupuncture therapy can be used as a non-drug treatment tool for patients with mild cognitive impairment (Ghafoor et al., 2019), and this direction has been a hot research topic in recent years. Therefore, this study aims to analyze the results of published clinical randomized controlled trials of acupuncture in patients with cognitive dysfunction in Alzheimer's disease by Cochrane systematic evaluation method. To evaluate the efficacy and safety of acupuncture in the treatment of cognitive impairment Alzheimer's disease.

## 2 Data

### 2.1 Nano row standard

#### 2.1.1 Research types

Comparing acupuncture and other treatments for Alzheimer's disease clinical curative effect and security of the randomized controlled trial (RCT) randomized controlled trial, in line with the AD diagnosis. The diagnostic criteria refer to the National Institute on Aging (NIA) and the Alzheimer's Association (AA) 2011 National Diagnostic Criteria for Alzheimer's Disease (McKhann et al., 2011), the National Stroke Institute for Neuropathic Language Disorders and the Association for AD and Related Disorders (NINCDS-ADRDA) (Tamaoka, 2011), Chinese Guidelines for the Diagnosis and Treatment of Alzheimer's Disease Dementia (2020 Edition) (OCDAD) (Tian et al., 2020), the Fifth edition of the Diagnostic and Statistical Manual of Mental Disorders (DSM-V-R) (Wang et al., 2015), the Sixth edition of the Diagnostic and Statistical Manual of Mental Disorders (DSM-IV-R) (American Psychiatric Association, 1994) of the American Psychiatric Association, The third edition of the Diagnostic and Statistical Manual of Mental Disorders (DSMIII-R) (Pichot, 1986) of the American Psychiatric Association, the 10th edition of the International Classification of Diseases (ICD-10) of the WHO.

#### 2.1.2 Intervention measure

The subjects in the intervention group were given acupuncture alone or combined with other treatments the same as the control group; the control group received conventional western medicine treatment; Treatment course ≥4 weeks.

#### 2.1.3 Ending indicators

The primary outcome indicators of this study were cognitive function evaluation, including: ① Simple Mental State Examination Scale (MMSE); ② Daily Living Ability Assessment Scale (ADL); ③ Alzheimer's Disease Cognitive Function Assessment Scale (ADAS-Cog); ④ TCM syndrome score (SDSD); ⑤ Montreal Cognitive Test (MoCA). The secondary outcome indicators were: ⑥ adverse reactions.

#### 2.1.4 Exclusion criteria

① Review, review, discussion and other non-clinical studies; ② Animal experiments or cell tissue studies; ③ Clinical studies of cases without randomized controlled trials or control groups; ④ Comparative study of disease group and non-disease group; ⑤ The treatment group combined with other TCM therapies, such as TCM therapy, massage therapy, acupoint application, foot bath therapy, etc.; ⑥ Self-cross-control study; ⑦ Clinical studies that cannot be traced back to original data or whose existing data are incomplete; ⑧ Clinical studies without access to original texts.

## 3 Methods

### 3.1 Literature search strategy

Chinese and English databases such as Pubmed, Embase, Cochrane Library Database, CBM, CNKI, VIP, and Wanfang were searched by computer, and randomized controlled clinical trials related to acupuncture in the treatment of Alzheimer's disease were searched. The time limit of retrieval is from the establishment of the database to July 31, 2022, and the retrieval method is a combination of subject words and free words. English search terms: acupuncture, Alzheimer's disease, randomized controlled trial; Chinese keywords included acupuncture, Alzheimer's disease, randomized controlled trials.

### 3.2 Literature screening and data extraction

Initial literatures were imported into EndNoteX9 software and a database of acupuncture therapy for Alzheimer's disease was established. Two researchers independently completed the work of inclusion, screening and information extraction by reading the title, abstract and full text. After the unilateral work was completed, cross-verification was conducted. If there is any disagreement during the process, the two researchers shall discuss and resolve it by themselves or request a third party to intervene to assist in judgment. After the inclusion and screening work was completed, the information extraction table was designed. The extraction contents mainly included: ① basic information included in the study; ② Baseline characteristics of subjects; ③ Intervention measures and outcome indicators included in the study; ④ Key elements of bias risk assessment.

### 3.3 Bias risk assessment of included studies

The quality of included studies was independently assessed by two researchers in accordance with the bias risk assessment tool recommended in the Cochrane Manual (Higgins et al., 2011), and cross-validation was conducted after unilateral work was completed. Bias risk assessment mainly includes random allocation method, allocation scheme hiding, blind method, integrity of outcome data, selective reporting of research results, and other sources of bias (baseline imbalance, claims of falsification). According to the results of the included literature, the judgment of “low risk of bias,” “high risk of bias,” and “unclear risk of bias” was made.

### 3.4 Statistical processing

Meta-analysis of the extracted information included in the study was performed by RevMan5.3 software. Binary data were represented by odds ratio (OR) and 95% confidence interval (95% CI), and measurement data were represented by mean difference (MD) and 95% confidence interval (95% CI). The results using standardized mean differences (standardized mean difference, SMD) and 95% confidence interval (95% CI). First, heterogeneity analysis was performed on the results of the included literatures. When *P* > 0.1 and *I*^2^ ≤ 50%, fixed effect model was used for meta-analysis. When *P* ≤ 0.1 and *I*^2^ > 50%, the sources of heterogeneity were analyzed. If the factors caused by patient age, gender, intervention measures, outcome indicators, etc., subgroup analysis or sensitivity analysis was carried out. After excluding the above interfering factors of clinical heterogeneity, the included results still showed heterogeneity, and random effects model was selected. If a certain outcome index included more than nine studies, funnel plots were drawn to analyze whether publication bias existed in the research results.

## 4 Result

### 4.1 Inclusion and screening

**Figure 1:**
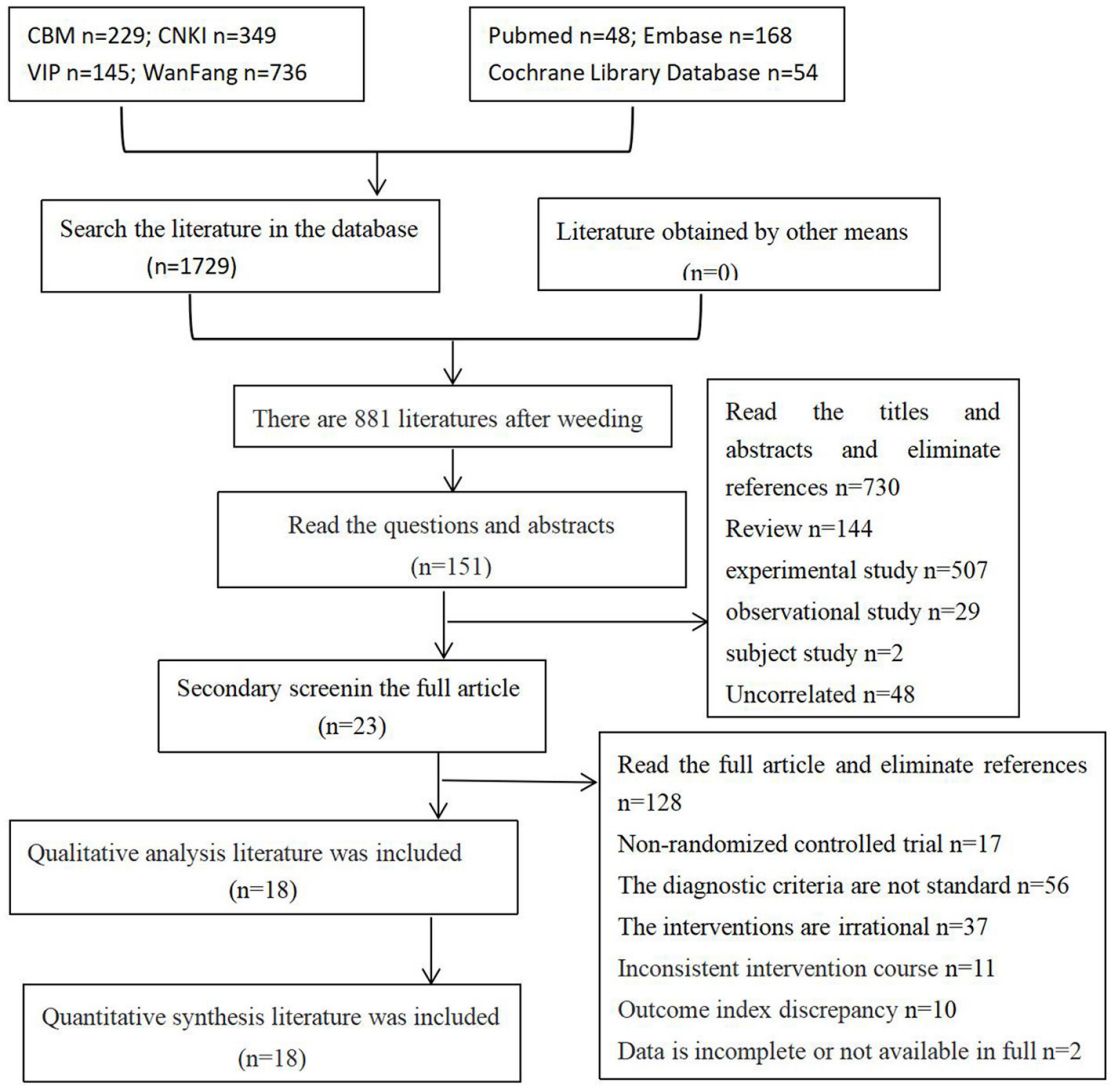
Literature screening process and results.

One thousand seven hundred twenty-nine related literatures were detected from the database, and 881 literatures were obtained after being imported into EndNote X9. After reading the title and abstract for preliminary screening, 151 literatures were obtained, and 18 literatures were obtained after reading the full text. Finally, 18 literatures were included, including 18 in Chinese and 0 in English. A total of 18 studies with 1,172 subjects were involved. See Figure 1 for details.

### 4.2 Basic features included in the study

After reading the title, abstract and full text, a total of 18 RCTS of acupuncture for AD were included according to the inclusion and exclusion criteria (Ouyang et al., 1999; Dong et al., 2002; Liu et al., 2008; Peng and Dong, 2009; Hu et al., 2010; Xia et al., 2010; Gu et al., 2014; Li, 2014; Lin et al., 2014; Yan et al., 2014; Yang, 2014; Zhang, 2014; Lin, 2016; Guan, 2017; Jia et al., 2017; He, 2018; Sun and Zhang, 2018; Feng et al., 2019), published from 1999 to 2020. CBM, CNKI, VIP, WanFang, Pubmed, Embase, Cochrane Library Database and other Chinese and English databases from the establishment of the database to December 31, 2022 were used as the sources of included literature search. A total of 1,172 subjects were included, and 595 cases were in the intervention group. There were 577 cases in the control group. See Table 1 for details.

**Table 1:** Basic features of the included literatures.

**Incorporate into study**	**Sample size**		**Intervening measure**		**Course of treatment (week)**	**Ending indicators**
	**T**	**C**	**T**	**C**		
Xia et al. (2010)	30	30	Donepezil + electroacupuncture	Donepezil	8	③④⑤
Feng et al. (2019)	17	16	Electric needle lily, Fengfu, God Court, Sun, Shang Yintang, big bell	Donepezil	12	①⑥
He (2018)	30	30	Donepezil + warm acupuncture	Donepezil	12	①④⑤
Sun and Zhang (2018)	55	55	Anying agent + acupuncture Baihui, Yongquan	Placebo	6	①
Guan (2017)	30	30	Donepezil + Chinese medicine Di Rong Zhizhi Granules + acupuncture	Donepezil + Dirong Zhizhi Granules	8	①②③
Jia et al. (2017)	35	36	Western medicine mold to agent + three jiao needle method	Donepezil	12	①②③⑤⑥
Lin (2016)	20	20	Donepezil + head needle	Donepezil	12	①③
Gu et al. (2014)	72	69	Acupuncture Shenting, Baihui, Fengchi, Wangu, Shanzhong, Zhongwan, Qi Sea, blood sea, Zusanli	Donepezil	8	①②③⑤⑥
Li (2014)	30	30	Donepezil + acupuncture	Donepezil	12	①②③
Lin et al. (2014)	18	18	Acupuncture Baihui, four Shencong, Neiguan, three Yin Jiao	Donepezil	12	①②③
Yan et al. (2014)	20	20	Needling Si Shencong, Shen Ting, Benshen, Shen Men, Taixi	Donepezil	4	①
Yang (2014)	30	30	Acupuncture Baihui, Sinshencong, Yintang, Hanging Zhong, Pishu, Shenshu, Taixi, Zusanli	Donepezil	4	①②④
Zhang (2014)	30	30	Needled Lily, Si Clergy, Yintang, Hanging Bell, Zhongwan, Fenglong and Zusanli	Donepezil	16	①②⑤
Hu et al. (2010)	40	40	Acupuncture Shanzhong, Zhongwan, Qi Sea, blood sea, Zusanli, Waiguan	No untreated	12	①②
Peng and Dong (2009)	28	28	Puzzle Jiannao Granules + acupuncture	Puzzle Jiannao granules	12	①②
Liu et al. (2008)	40	40	Sniff three needling	Duxil	10	①
Dong et al. (2002)	10	11	Acupuncture Baihui, Dazhui, Shenshu, Shenmen, neiguan, Sanyinjiao, Sishencong, Fengchi, Taixi, Zusanli, Fenglong, Taichong	Psychological counseling, daily life guidance	12	①②
Ouyang et al. (1999)	16	14	Acupuncture Baihui, Sishentax, Shenshu, Taichong, Guanyuan, Sanyinjiao, Zusanli	Nimodipine	8	②

T: intervention group; C: control group; ① MMSE; ② ADL; ③ ADAS-Cog; ④ SDSD; ⑤ MoCA; ⑥ Adverse reactions.

### 4.3 Bias risk assessment

**Figure 2:**
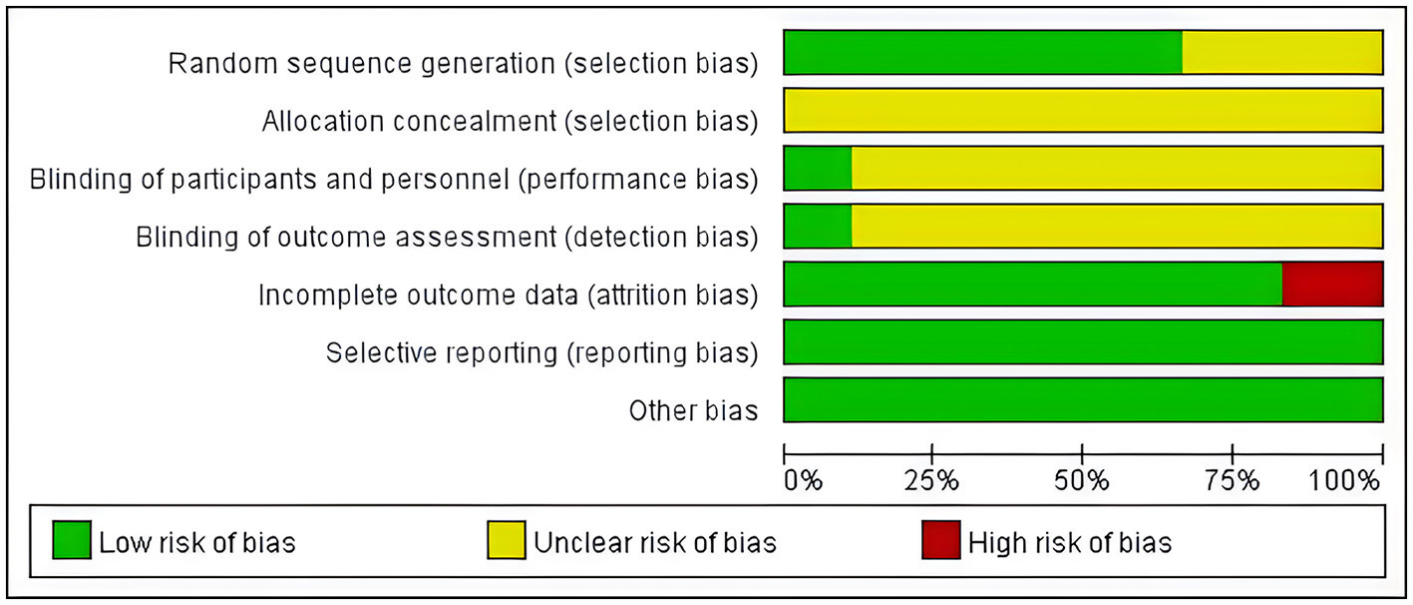
Bias risk analysis of included studies.

**Figure 3:**
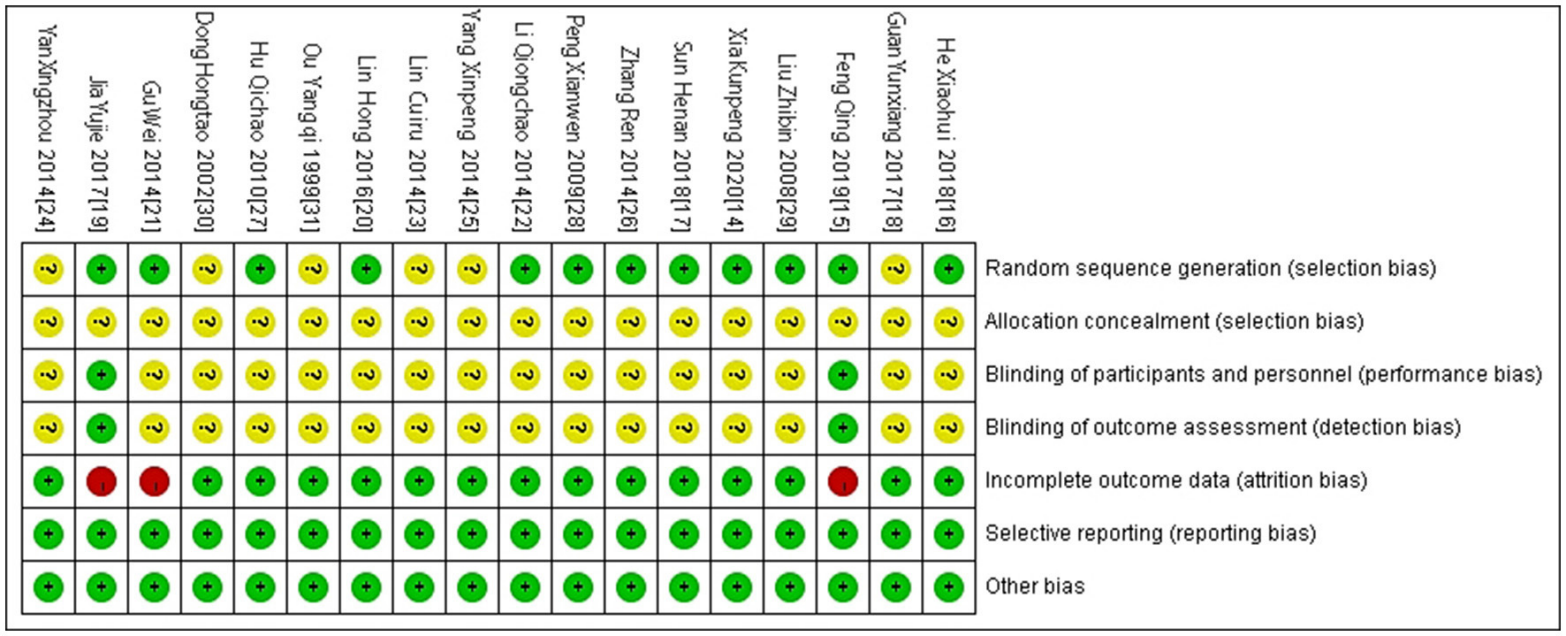
Individual bias risk analysis included in the study.

The quality of included studies was assessed using bias risk assessment tools recommended in the Cochrane Manual. Eighteen studies all mentioned “random” grouping, among which nine studies adopted the random number table method (Liu et al., 2008; Gu et al., 2014; Yang, 2014; Zhang, 2014; Lin, 2016; Guan, 2017; Jia et al., 2017; He, 2018; Sun and Zhang, 2018), two studies adopted the random allocation of envelope number method (Xia et al., 2010; Feng et al., 2019), two studies adopted the lottery method (Peng and Dong, 2009; Lin et al., 2014), and one study adopted the survey sequence grouping method (Hu et al., 2010). The four studies did not describe the random grouping method and the specific implementation process in detail (Ouyang et al., 1999; Dong et al., 2002; Lin et al., 2014; Yan et al., 2014). None of the included studies reported the assignment hiding scheme. Two studies (Jia et al., 2017; Feng et al., 2019) adopted “double-blind” in the process, and the remaining studies did not report the implementation of the blind method in detail. All the included studies had baseline comparability. Data shedding occurred in three studies (Gu et al., 2014; Jia et al., 2017; Feng et al., 2019) and the reasons for shedding were clear, while the remaining studies had complete data. According to the research results, the above six aspects were judged as high risk, low risk and unclear. The bias of risk assessment and efficacy outcome indexes in the included 18 studies was stable. See Figures 2, 3 for details.

### 4.4 Results of meta-analysis

#### 4.4.1 MMSE score

**Figure 4:**
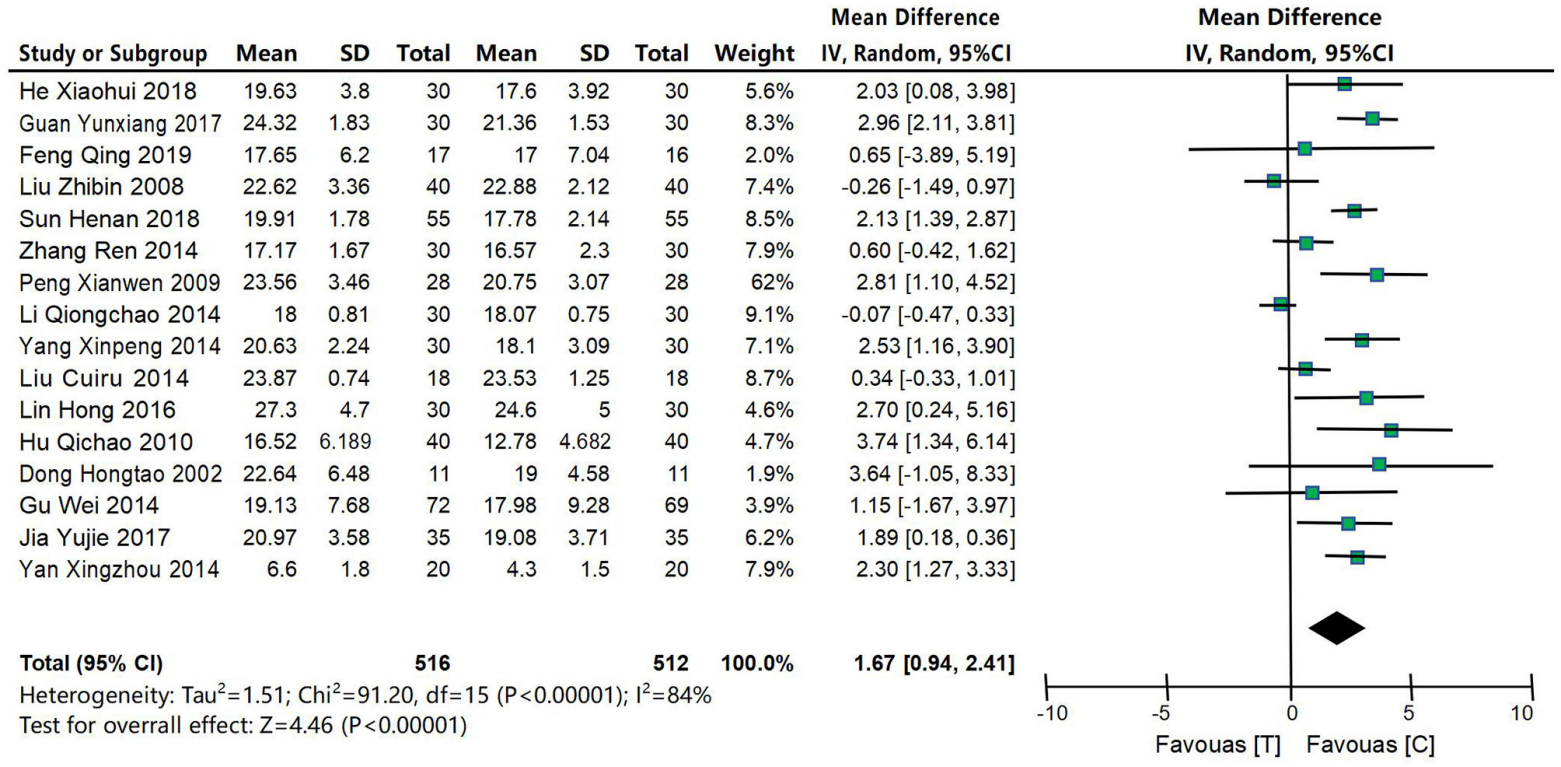
Forest map of MMSE score meta-analysis between acupuncture intervention group and control group. The results of meta-analysis showed that the improvement effect of MMSE scores in the acupuncture intervention group was significantly better than that in the control group, and the difference was statistically significant [MD = 1.67, 95% CI (0.94, 2.41), *P* < 0.00001].

Sixteen studies (Dong et al., 2002; Liu et al., 2008; Peng and Dong, 2009; Hu et al., 2010; Gu et al., 2014; Li, 2014; Lin et al., 2014; Yan et al., 2014; Yang, 2014; Zhang, 2014; Lin, 2016; Guan, 2017; Jia et al., 2017; He, 2018; Sun and Zhang, 2018; Feng et al., 2019) were included to compare MMSE scores between the acupuncture intervention group and the control group, as shown in Figure 4. XX was a continuous variable, and MD and 95% confidence interval were used for analysis. Heterogeneity test results were as follows: *P* < 0.00001, *I*^2^ = 84%, indicating large heterogeneity in the included studies, so the random effects model was used for meta-analysis. The results of meta-analysis showed that the improvement effect of MMSE scores in the acupuncture intervention group was significantly better than that in the control group, and the difference was statistically significant [MD = 1.67, 95% CI (0.94, 2.41), *P* < 0.00001], as shown in Figure 4.

#### 4.4.2 ADL score

**Figure 5:**
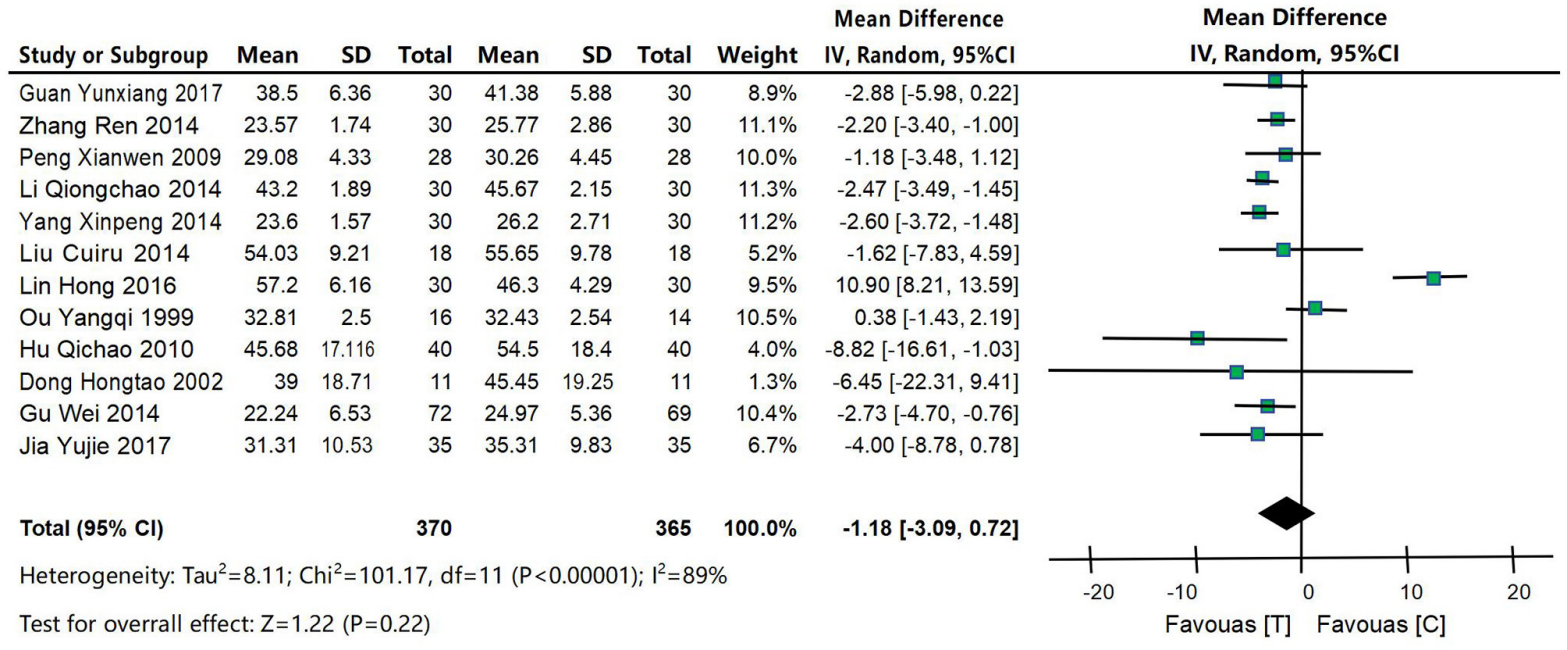
Forest map of ADL score meta-analysis between acupuncture intervention group and control group. The results of meta-analysis showed that: the improvement of ADL scores in the acupuncture intervention group tended to be better than that in the control group, or had clinical significance, but did not reach the statistical difference standard [MD = −1.18, 95% CI (−3.09, 0.72), *P* = 0.22].

Twelve studies (Ouyang et al., 1999; Dong et al., 2002; Peng and Dong, 2009; Hu et al., 2010; Gu et al., 2014; Li, 2014; Lin et al., 2014; Yang, 2014; Zhang, 2014; Lin, 2016; Guan, 2017; Jia et al., 2017) were included to compare ADL scores between the acupuncture intervention group and the control group, as shown in Figure 5. XX was a continuous variable, and MD and 95% confidence interval were used for analysis. The heterogeneity test results were *P* = 0.22 and *I*^2^ = 89%, indicating that the heterogeneity of included studies was large, so the random effects model was used for meta-analysis. The results of meta-analysis showed that: the improvement of ADL scores in the acupuncture intervention group tended to be better than that in the control group, or had clinical significance, but did not reach the statistical difference standard [MD = −1.18, 95% CI (−3.09, 0.72), *P* = 0.22], as shown in Figure 5.

#### 4.4.3 ADAS-Cog scoring

**Figure 6:**
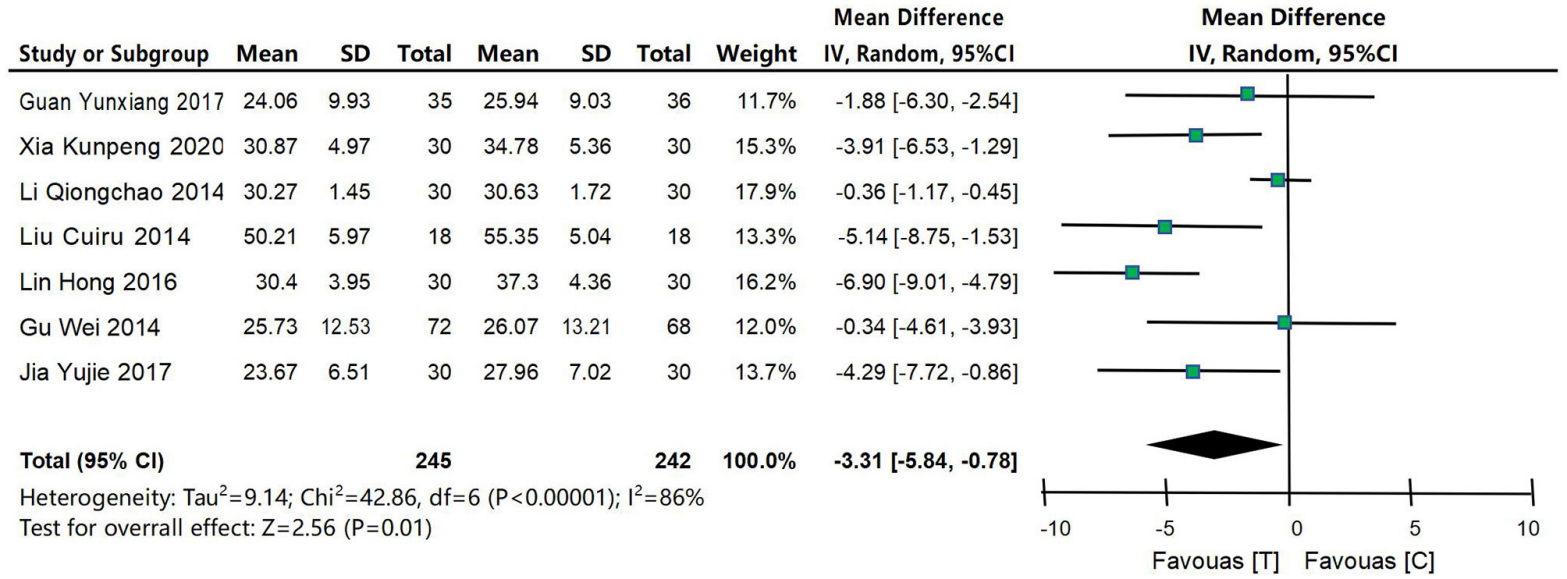
Forest map of ADAS-Cog score meta-analysis between acupuncture intervention group and control group. The results of meta-analysis showed that: the results showed that the improvement of ADAS-Cog scores in the acupuncture intervention group was significantly better than that in the control group, the difference was statistically significant [MD = −3.31, 95% CI (−5.84, −0.78), *P* = 0.01].

Seven studies (Xia et al., 2010; Gu et al., 2014; Li, 2014; Lin et al., 2014; Lin, 2016; Guan, 2017; Jia et al., 2017) were included to compare ADAS-Cog scores between the acupuncture intervention group and the control group, as shown in Figure 6. XX was a continuous variable, and MD and 95% confidence interval were used for analysis. The heterogeneity test results were *P* = 0.01 and *I*^2^ = 86%, indicating that the heterogeneity of the included studies was large, so the random effects model was used for meta-analysis. The results of meta-analysis showed that: the results showed that the improvement of ADAS-Cog scores in the acupuncture intervention group was significantly better than that in the control group, the difference was statistically significant [MD = −3.31, 95% CI (−5.84, −0.78), *P* = 0.01].

#### 4.4.4 SDSD score

**Figure 7:**
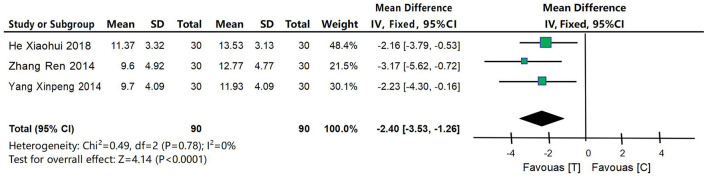
Forest map of meta-analysis of SDSD scores between acupuncture intervention group and control group. The results of meta-analysis showed that: the results showed that the improvement of SDSD scores in the acupuncture intervention group was significantly better than that in the control group, with statistical significance [MD = −2.40, 95% CI (−3.53, −1.26), *P* < 0.0001].

Three studies (Yang, 2014; Zhang, 2014; He, 2018) were included to compare SDSD scores between the acupuncture intervention group and the control group, as shown in Figure 7. XX was a continuous variable, and MD and 95% confidence interval were used for analysis. Heterogeneity test results were *P* < 0.0001 and *I*^2^ = 0%, indicating small heterogeneity in the included studies, so fixed effect model was used for meta-analysis. The results of meta-analysis showed that: the results showed that the improvement of SDSD scores in the acupuncture intervention group was significantly better than that in the control group, with statistical significance [MD = −2.40, 95% CI (−3.53, −1.26), *P* < 0.0001].

#### 4.4.5 MoCA score

**Figure 8:**
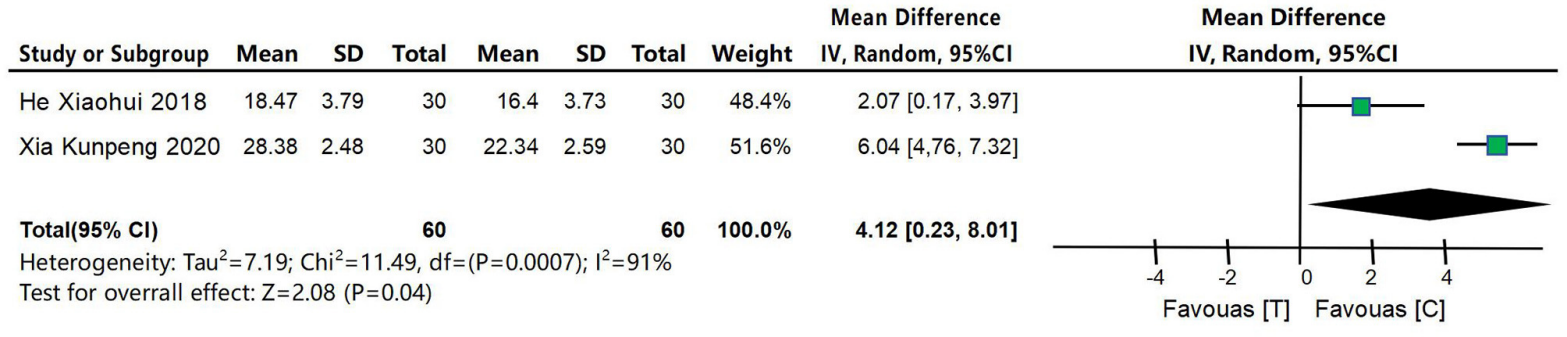
Forest map of MoCA score meta-analysis between acupuncture intervention group and control group. The results of meta-analysis showed that the improvement of MoCA score in acupuncture intervention group was better than that in control group, and the difference was statistically significant [MD = 4.80, 95% CI (3.74, 5.86), *P* = 0.04].

Two studies (Xia et al., 2010; He, 2018) were included to compare MoCA scores between the acupuncture intervention group and the control group, as shown in Figure 8. MoCA score was a continuous variable and was analyzed using MD and 95% confidence interval. The heterogeneity test results were *P* = 0.04 and *I*^2^ = 91%, indicating that the heterogeneity of the included studies was large, so the random effects model was used for meta-analysis. The results of meta-analysis showed that the improvement of MoCA score in acupuncture intervention group was better than that in control group, and the difference was statistically significant [MD = 4.80, 95% CI (3.74, 5.86), *P* = 0.04].

#### 4.4.6 Adverse reaction

**Figure 9:**
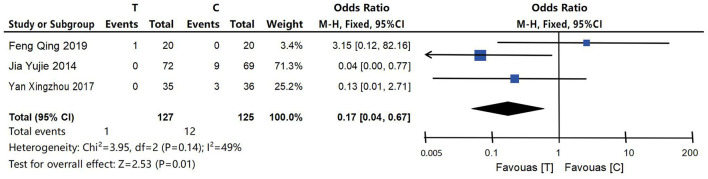
Meta-analysis forest map of adverse reactions included in the study. No serious adverse reactions related to acupuncture were found in the acupuncture intervention group, and the incidence and severity of adverse reactions were lower than those in the control group, with statistical significance [OR = 0.17, 95% CI (0.04, 0.67), *P* = 0.01].

In the 18 studies included, the occurrence of adverse reactions was a dichotomous variable, which was analyzed using OR and 95% confidence interval. Among them, three studies (Gu et al., 2014; Jia et al., 2017; Feng et al., 2019) described the incidence and specific conditions of adverse reactions during the test in detail. In one study (Feng et al., 2019), a patient in the acupuncture intervention group developed local hematoma after the eighth treatment, and the hematoma dissipated spontaneously after 4-days without special treatment. In one study (Gu et al., 2014), one patient in the control group suffered from stomach discomfort, while 8 patients suffered from adverse reactions such as loss of appetite, nausea, diarrhea and insomnia without further treatment. In one study (Jia et al., 2017), three patients in the control group showed adverse reactions such as nausea, loss of appetite, diarrhea, constipation, fatigue, etc. Two patients stopped taking medication, and one patient's reaction disappeared after continuing to take medication. No serious adverse reactions related to acupuncture were found in the acupuncture intervention group, and the incidence and severity of adverse reactions were lower than those in the control group, with statistical significance [OR = 0.17, 95% CI (0.04, 0.67), *P* = 0.01], as shown in Figure 9.

#### 4.4.7 Publication bias

**Figure 10:**
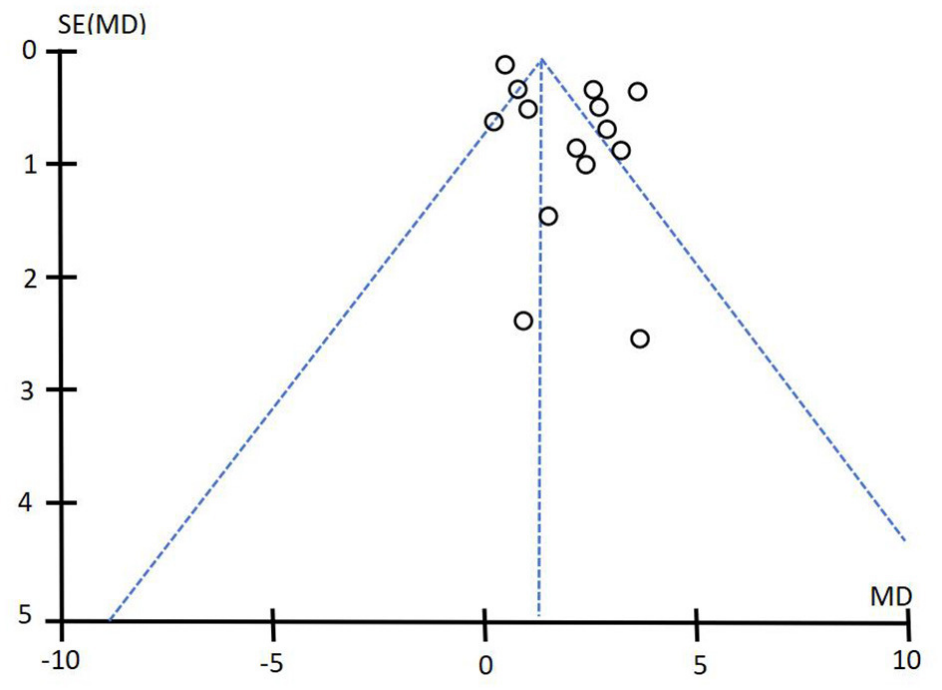
Funnel diagram of MMSE score.

**Figure 11:**
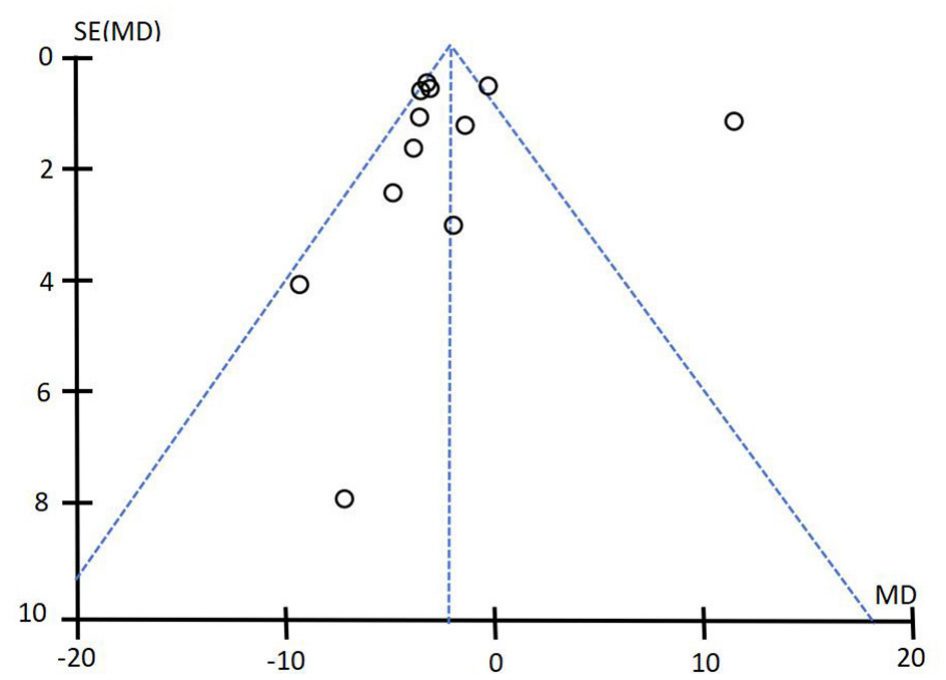
Funnel diagram of ADL score. The results showed that the funnel plots of MMSE and ADL were asymmetrical, suggesting that publication bias may exist in this systematic evaluation. See Figure 10 and this figure for details.

In this systematic evaluation, 16 studies on MMSE score and 12 studies on ADL score were included in acupuncture intervention group and control group. Funnel plots were drawn for MMSE score and ADL score to detect publication bias. The results showed that the funnel plots of MMSE and ADL were asymmetrical, suggesting that publication bias may exist in this systematic evaluation. See Figures 10, 11 for details.

## 5 Discussion

Alzheimer's disease belongs to the categories of “dementia,” “stupidness,” and “depression syndrome” in traditional Chinese medicine. The disease is located in the brain and is closely related to the disorders of liver, heart, spleen and kidney function. The pathogenesis of Alzheimer's disease is mainly based on deficiency, phlegm and blood stasis. Or mood failure, long stagnation does not understand, wood sheng soil decline, gather wet phlegm, phlegm turbidity Mengqiao; or after stroke, trauma, qi stagnation and blood stasis, brain collateral stasis, brain qi impassability, brain qi, viscera qi is not connected. Cognitive impairment is the most common complaint of patients in the pre-dementia stage and the key clinical manifestation in the continuous progressive development of AD. The stage of dementia in AD patients can be divided according to the degree of cognitive impairment. Currently, there is no specific prevention or treatment for AD patients, and clinically applied acetylcholinesterase inhibitors and memantine can only slow down the progression of the disease (Koseoglu, 2019), while corresponding supportive treatment and symptomatic treatment can be taken for the complications of severe patients, which cannot achieve the expectation of preventing the development of the disease or promoting the recovery of the disease.

At present, there is no definite conclusion on the pathogenesis of AD, and there are many theories. Among them, β-amyloid waterfall hypothesis is highly recognized, which suggests that the imbalance between the production and clearance of β-amyloid protein in the brain of AD patients, the aggregation to form age spots, and the mitochondrial damage induced by neurotoxicity (Yuyama and Igarashi, 2017) are the initial events of neuronal degeneration and dementia. Acupuncture can reduce the deposition of β-amyloid protein in the brain of AD patients by affecting the cleavage and degradation pathways, regulate the functional activities and connectivity of specific cognitively related regions (Zheng et al., 2018), and thus improve the spatial learning and memory ability of the subjects (Heneka et al., 2005; Jha et al., 2015; Wang et al., 2017, 2018; Zhang et al., 2017; Tang et al., 2018, 2019, 2020; Yang et al., 2018; Jiang et al., 2019; Khan et al., 2019). Another theory with wide influence is the theory of abnormal function of microtubule-associated proteins. Studies have shown that Tau hyperphosphorylation is positively correlated with the degree of dementia in AD patients (Berg et al., 1998), that is, over-phosphorylated tau protein affects the stability of neuronal callus tubulin and forms tangles, thus destroying the normal function of neurons and synapses. However, acupuncture can change the activity of other protein kinases to affect tau protein phosphorylation level in AD patients (Xu et al., 2010) and delay the aggregation process of Tau protein that is over-phosphorylated (Yan et al., 2014). In recent years, some scholars have also proposed the insulin hypothesis, that is, diabetes is a high risk factor for AD (Silva et al., 2019), the glucose metabolism and blood flow in the cognitively related brain area of AD patients have significant changes (Dukart et al., 2013), and the reversal of cerebral glucose metabolism rate and insulin resistance can promote the recovery of learning and memory ability in animal models (Kang et al., 2017). Studies have shown that acupuncture can improve brain glucose metabolism and blood supply in AD patients by affecting the neuroendocrine system, increasing glucose metabolism and alleviating insulin resistance (Ding et al., 2019; Fanibunda et al., 2019; Shu et al., 2020). In addition, there are many hypotheses such as vascular factors, gene mutation, oxidative stress, inflammatory mechanism and neurogenesis.

The onset of AD is insidious, with cognitive impairment and decline in activities of daily living as the main manifestations in the early stage of the disease. In this study, we searched CBM, CNKI, VIP, Wanfang, Pubmed, Embase, Cochrane Library Database and other Chinese and English databases with the key words of “acupuncture,” “acupuncture,” “Alzheimer's disease,” and “AD.” The literatures related to RCTS on the efficacy and safety of acupuncture in the treatment of cognitive impairment in AD from the establishment of the database to December, 2022 were screened, and the outcome indicators such as MMSE, ADL, ADAS-Cog, SDSD and MoCA were comprehensively analyzed. A total of 18 RCTS were included, evaluating 1,172 subjects. The results showed that: acupuncture and acupuncture combined with other treatments were superior to the control group in improving the scores of MMSE, ADAS-Cog, SDSD and MoCA (*P* < 0.05). The improvement of ADL score in the experimental group was better than that in the control group, or had clinical significance, but did not reach the statistical difference standard (*P* = 0.22). Security analysis shows: compared with the control group, the incidence of adverse reactions in acupuncture intervention group was lower and the safety was better. Simple medication, acupuncture and medication + acupuncture are all effective in the treatment of AD cognitive dysfunction. Acupuncture + Chinese medicine + western medicine has the best effect, and acupuncture + medication is more effective than simple acupuncture or medication. Compared with drug therapy, simple acupuncture has better effects in improving MMSE, ADL, ADAS-cog scores and continuous treatment effect.

As for the efficacy of acupuncture in treating AD, nine of the included studies reported on the overall effectiveness rate of acupuncture, revealing that compared to the drug treatment group, acupuncture demonstrated a higher total effective rate in AD treatment. Furthermore, cognitive function improvement in AD patients can be evaluated using MMSE, ADL, ADAS-Cog, SDSD and MoCA scores. The results indicated that the acupuncture intervention group exhibited superior improvements in MMSE, ADL, ADAS-Cog, SDSD and MoCA scores compared to the control group or displayed a significant trend toward efficacy. However, due to limitations such as literature quality and sample size constraints within this study's inclusion criteria; it is currently not possible to reasonably evaluate the impact of acupuncture on MMSE, ADL, ADAS-Cog, SDSD, and MoCA scores in patients with Alzheimer's disease. Although there is insufficient evidence at present (Zhou et al., 2015; Huang et al., 2019) to confirm its safety as a treatment for Alzheimer's disease (AD), acupuncture has been widely utilized in clinical practice over recent years with few reports regarding related side effects. The overall incidence of adverse reactions observed was 16.67%, out of which only 5.56% were attributed specifically to acupuncture therapy; these included two cases where side effects like nausea, diarrhea, and insomnia were caused by drugs administered within the control group and one case involving local hematoma formation after an acupuncture session. To some extent, the incidence of adverse reactions associated with acupunctural treatments for Alzheimer's disease was low, mild or even characterized by good efficacy combined with minimal side effects.

The treatment time span of the included studies was 4–16 weeks, and the treatment and observation time was short, among which four studies (Hu et al., 2010; Yang, 2014; Zhang, 2014; Lin, 2016) had a treatment course of 4 weeks, four studies (Ouyang et al., 1999; Xia et al., 2010; Li, 2014; Guan, 2017) had a treatment course of 8 weeks, seven studies (Dong et al., 2002; Peng and Dong, 2009; Lin et al., 2014; Yan et al., 2014; Jia et al., 2017; He, 2018; Feng et al., 2019) had a treatment course of 12 weeks, and one study (Sun and Zhang, 2018) had a treatment course of six weeks. The treatment course of one study (Liu et al., 2008) was 10 weeks, and the treatment course of another study (Gu et al., 2014) was 16 weeks. The acupuncture operation requirements included in the study were all conventional acupuncture, that is, flat acupuncture, oblique acupuncture and straight acupuncture should be performed according to the body parts of the acupoints. Thirteen studies (Pichot, 1986; Liu et al., 2008; Peng and Dong, 2009; Hu et al., 2010; Xia et al., 2010; Higgins et al., 2011; Gu et al., 2014; Li, 2014; Yan et al., 2014; Zhang, 2014; Lin, 2016; He, 2018; Feng et al., 2019) took obtaining qi as the criterion for the effectiveness of acupuncture. In 13 studies (Ouyang et al., 1999; Dong et al., 2002; Liu et al., 2008; Peng and Dong, 2009; Xia et al., 2010; Li, 2014; Lin et al., 2014; Yan et al., 2014; Zhang, 2014; Guan, 2017; He, 2018; Sun and Zhang, 2018; Feng et al., 2019), the effects of qi were enhanced by lifting, twisting and switching to supplementing or purging. Fifteen included studies detailed the duration of needle retention after acupuncture, and nine studies (Ouyang et al., 1999; Peng and Dong, 2009; Gu et al., 2014; Li, 2014; Lin et al., 2014; Lin, 2016; Guan, 2017; He, 2018; Sun and Zhang, 2018; Feng et al., 2019) kept needles for 30 min after acupuncture. Five studies (Dong et al., 2002; Xia et al., 2010; Yan et al., 2014; Yang, 2014; Zhang, 2014) retained needles for 40 min after acupuncture, one study (Liu et al., 2008) retained needles for 60 min after acupuncture, and the remaining studies did not provide a clear explanation on the length of retained needles after acupuncture. In the included seven studies, acupuncture process was accompanied by other auxiliary means, among which five studies (Ouyang et al., 1999; Dong et al., 2002; Xia et al., 2010; Lin et al., 2014; Feng et al., 2019) used electroacupuncture as auxiliary means of acupuncture, with variable waveform, frequency of electroacupuncture of 10 Hz/50 Hz, intensity of 0.5–5.0 mA, and patient tolerance as the degree. One study (He, 2018) used warm acupuncture as an auxiliary acupuncture method, and one study (Peng and Dong, 2009) used ginger moxibustion as an auxiliary acupuncture method.

## 6 Limitations

The random assignment process, assignment concealability, and blind-causing description of participants, investigators, and evaluators are problematic due to defects in the experimental methods and reported results used in the inclusion RCTS; Moreover, the effectiveness of therapeutic measures is potentially correlated with factors such as acupoint selection, acupuncture timing, acupuncture technique, acupuncture mode, administration mode, length of treatment course, etc., so there is a certain publication bias in this study. Its limitations mainly include: There are few RCT studies on the treatment of AD cognitive dysfunction with acupuncture, and the sample size is small; There were differences in baseline level, acupuncture course, follow-up work and drug dosage among included studies; Some included studies did not specifically describe random method, blind method and assignment hiding scheme.

## 7 Conclusion

In conclusion, the present study showed that acupuncture is as effective as or even better than existing medical treatments in treating cognitive impairment in AD. Acupuncture is helpful to improve the cognitive function and self-care ability of patients, and it is safer in clinical application. However, the results of this study are affected by the limited number of RCTS evaluating the efficacy of acupuncture and the bias of the included studies. Therefore, future research should focus on exploring the effects of acupuncture on cognitive impairment in Alzheimer's disease in delaying cognitive decline, improving daily function, reducing mortality and other functional deficits, and conduct more high-quality and large-sample RCT studies on the synergistic effect of acupuncture, drug therapy and psychological counseling. In order to provide stronger evidence to support the efficacy and safety of acupuncture treatment.

## Data availability statement

The original contributions presented in the study are included in the article/Supplementary material, further inquiries can be directed to the corresponding author.

## Author contributions

RG: Data curation, Writing – original draft, Writing – review & editing. XS: Supervision, Validation, Writing – review & editing, Writing – original draft. JE: Methodology, Writing – review & editing. JZ: Data curation, Writing – review & editing. JL: Data curation, Writing – review & editing. YN: Data curation, Writing – review & editing.
