# Supplementary material for: Efficacy and safety of acupuncture for cognitive impairment in Alzheimer's disease: a systematic review and meta-analysis
Source: Front Dement. 2024 Jul 3;3:1380221. doi: 10.3389/frdem.2024.1380221 (PMC11285646; doi:10.3389/frdem.2024.1380221)
Supplement: Supplementary file 1 [file Table_1.docx]

***Supplementary table S1:* The details of search terms and literature search strategy**

Take searching PubMed as an example, the search terms and strategies are as follows:

| #1Acupuncture [MeSH Terms]  #2(((((((((Acupuncture Treatment[Title/Abstract]) OR (Acupuncture Treatments[Title/Abstract])) OR (Treatment, Acupuncture[Title/Abstract])) OR (Therapy, Acupuncture[Title/Abstract])) OR (Pharmacoacupuncture Treatment[Title/Abstract])) OR (Treatment, Pharmacoacupuncture[Title/Abstract])) OR (Pharmacoacupuncture Therapy[Title/Abstract])) OR (Therapy, Pharmacoacupuncture[Title/Abstract])) OR (Acupotomy[Title/Abstract])) OR (Acupotomies[Title/Abstract])  #3#1 OR #2  #4Alzheimer's disease[MeSH Terms]  #5 Alzheimer[MeSH Terms]  #6((Alzheimer's disease[Title/Abstract]) OR (Alzheimer[Title/Abstract]))  #7#4 OR #5 OR #6  #8((randomized controlled trial[Publication Type]) OR (randomized[Title/Abstract])) OR (placebo[Title/Abstract])  #9 #3 AND#7 AND #8 |
| --- |

***Supplementary table S2:*The composition and effect of Chinese patent medicine in this article**

First, Yizhi Jiannao granules

1. Drug composition: Epimedium, Polygonum multiflorum, leech, Angelica sinensis, Suoyang, Chuanduan, Tianqi, Paeony, Acanthopanax, Cypress seed

2. Production unit: Hunan Dekang Pharmaceutical Co., LTD., production lot No. 20130202

3. Usage and Dosage: 5.5g per pack. 1 pack each time, 3 times a day.

4. Efficacy: spleen tonifying kidney, brain health

Second, Dirong Zhizhi granules

1. Drug composition: ripe ground, Cistanche deserticola, Euphorbia officinalis, Codonopsis codonopsis, Ophiopogon, dodder, jujube kernel, Radix polygala, Radix dragon, orange peel, poria, Salviorrhiza, calamus, licorice

2. Production unit: The First Affiliated Hospital of Henan University of Chinese Medicine

3. Usage and Dosage: 1 dose per day, divided into 2 times.

4. Efficacy: tonifying liver and kidney, puzzle brain
